# Early visual alterations in individuals at-risk of Alzheimer’s disease: a multidisciplinary approach

**DOI:** 10.1186/s13195-023-01166-0

**Published:** 2023-01-24

**Authors:** Inés López-Cuenca, Alberto Nebreda, Alejandra García-Colomo, Elena Salobrar-García, Jaisalmer de Frutos-Lucas, Ricardo Bruña, Ana I. Ramírez, Federico Ramirez-Toraño, Juan J. Salazar, Ana Barabash, Pedro Gil, Fernando Maestú, José M. Ramírez, Rosa de Hoz

**Affiliations:** 1 Ramon Castroviejo Institute for Ophthalmic Research, Complutense University of Madrid, 28040 Madrid, Spain; 2 Health Research Institute of the Hospital Clínico San Carlos (IdISSC), 28040 Madrid, Spain; 3 Center for Cognitive and Computational Neuroscience, Complutense University of Madrid, 28223 Pozuelo de Alarcón, Spain; 4 Department of Experimental Psychology, Cognitive Psychology and Speech & Language Therapy, Complutense University of Madrid, 28223 Pozuelo de Alarcón, Spain; 5 Department of Immunology, Ophthalmology and ENT, Faculty of Optics and Optometry, University of Madrid, 28040 Madrid, Spain; 6 Centre for Precision Health, Edith Cowan University, Joondalup, WA 6027 Australia; 7 Department of Psychology, Nebrija University, 28015 Madrid, Spain; 8 Department of Industrial Engineering & IUNE, University of La Laguna, 38200 San Cristobal de La Laguna, Spain; 9 Department of Radiology, Complutense University of Madrid, 28040 Madrid, Spain; 10 Department of Endocrinology and Nutrition, Hospital Clínico San Carlos, 28040 Madrid, Spain; 11 Centre for Biomedical Research Network On Diabetes and Associated Metabolic Diseases, 28029 Madrid, Spain; 12 Department of Medicine II, School of Medicine, Complutense University of Madrid, 28040 Madrid, Spain; 13 Department of Medicine, School of Medicine, Complutense University of Madrid, 28040 Madrid, Spain; 14 Memory Unit, Geriatrics Service, Hospital Clínico San Carlos, 28040 Madrid, Spain; 15 Biomedical Research Networking Center in Bioengineering, Biomaterials and Nanomedicine, 28029 Madrid, Spain; 16 Department of Immunology, Ophthalmology and ENT, Faculty of Medicine, Complutense University of Madrid, 28040 Madrid, Spain

**Keywords:** Alzheimer’s disease, Magnetoencephalography, Optical coherence tomography, At risk for AD, Visual function

## Abstract

**Background:**

The earliest pathological features of Alzheimer’s disease (AD) appear decades before the clinical symptoms. The pathology affects the brain and the eye, leading to retinal structural changes and functional visual alterations. Healthy individuals at high risk of developing AD present alterations in these ophthalmological measures, as well as in resting-state electrophysiological activity. However, it is unknown whether the ophthalmological alterations are related to the visual-related electrophysiological activity. Elucidating this relationship is paramount to understand the mechanisms underlying the early deterioration of the system and an important step in assessing the suitability of these measures as early biomarkers of disease.

**Methods:**

In total, 144 healthy subjects: 105 with family history of AD and 39 without, underwent ophthalmologic analysis, magnetoencephalography recording, and genotyping. A subdivision was made to compare groups with less demographic and more risk differences: 28 high-risk subjects (relatives/APOEɛ4 +) and 16 low-risk (non-relatives/APOEɛ4 −).

Differences in visual acuity, contrast sensitivity, and macular thickness were evaluated. Correlations between each variable and visual-related electrophysiological measures (M100 latency and time–frequency power) were calculated for each group.

**Results:**

High-risk groups showed increased visual acuity. Visual acuity was also related to a lower M100 latency and a greater power time–frequency cluster in the high-risk group. Low-risk groups did not show this relationship. High-risk groups presented trends towards a greater contrast sensitivity that did not remain significant after correction for multiple comparisons. The highest-risk group showed trends towards the thinning of the inner plexiform and inner nuclear layers that did not remain significant after correction. The correlation between contrast sensitivity and macular thickness, and the electrophysiological measures were not significant after correction. The difference between the high- and low- risk groups correlations was no significant.

**Conclusions:**

To our knowledge, this paper is the first of its kind, assessing the relationship between ophthalmological and electrophysiological measures in healthy subjects at distinct levels of risk of AD. The results are novel and unexpected, showing an increase in visual acuity among high-risk subjects, who also exhibit a relationship between this measure and visual-related electrophysiological activity. These results have not been previously explored and could constitute a useful object of research as biomarkers for early detection and the evaluation of potential interventions’ effectiveness.

**Supplementary Information:**

The online version contains supplementary material available at 10.1186/s13195-023-01166-0.

## Background

Alzheimer’s disease (AD), the most prevalent cause of dementia, is characterized by the accumulation of amyloid-beta (Aβ) deposits and aggregates of hyperphosphorylated tau protein. This accumulation begins decades before a clinical diagnosis of the disease can be made and is followed by synaptic and neural loss, and, later, cognitive decline [1–3]. A growing body of evidence suggests that AD is associated with retinal pathology and visual dysfunction [4, 5]. For example, Aβ protein not only accumulates in the brain, but is also found in retinal deposits located in different layers [6]. The retina, part of the central nervous system, is composed of several layers of interconnected neurons. The transparent nature of the eye provides a unique opportunity for studying the effect of diseases on the central nervous system through objective, quantitative measurements, using in vivo real-time images of ocular structures like optical coherence tomography (OCT), a non-invasive imaging technique that detects variations in the inner and outer layers of the retina [7]. Changes in the structure of the retina have been found in AD patients using this technique [8]. These structural differences are accompanied by functional alterations in visual acuity, contrast sensitivity, and the tritan axis of color perception [7, 8].

Whether these retinal disruptions found in AD patients appear at the preclinical stage of the disease has been scarcely studied. Individuals at high risk for developing dementia are good targets for investigating these potential early disruptions.

Having a first-degree relative with the pathology and being a carrier of the ɛ4 allele of the apolipoprotein E (*APOE*) gene are, second only to the subject’s age, the main risk factors for the disease [9, 10]. So much so that first-degree relatives who are ɛ3/ɛ4 carriers present a lifetime risk of 46.1% and ɛ4/ɛ4 carriers of up to 61.4%, while the risk for first-degree relatives carrying ɛ3/ɛ3 alleles drops to 29.2% [11, 12]. Recent studies have found early and slight changes in the thickness (around ± 7 µm) of some macular regions of healthy individuals at high risk of developing dementia [13]. Therefore, this population at risk of developing the disease is of great interest for the identification of early neurophysiological characteristics of the disease that could act as early biomarkers and allow the implementation of prevention treatments, clinical trials, and pharmacological and non-pharmacological interventions.

It has not yet been demonstrated whether these macular changes have an impact on visual processing at a cortical level. Magnetoencephalography (MEG) is a non-invasive technique that measures the electromagnetic activity produced by the brain and has been repeatedly used to evaluate the components of visually evoked fields [14, 15]. Moreover, it is capable of correctly identifying patients with mild cognitive impairment, considered a prodromal stage of AD [16, 17]. Additionally, MEG is capable of detecting alterations in subjects at high risk of developing the disease in functional measures, such as relative power and functional connectivity [18–23]. Previous studies have found associations between visual evoked potentials and visual functioning in patients with macular disease; specifically, an association between a higher visual acuity and a lower P100 latency and an increased P100 amplitude was found in those patients [24].

Given the lack of studies assessing the link between early alterations in visual functioning and retinal structure and their relationship with visual cortical processing in AD, we evaluated subjects at distinct levels of risk of developing the pathology. For this purpose, we conducted an ophthalmological evaluation which measured visual acuity and contrast sensitivity, a face visualization task measured with MEG to evaluate the M100 latency and time–frequency power, and retinal imaging with OCT to evaluate retinal thickness. In the present work, we seek to answer three research questions: First, to assess whether the visual acuity of subjects at higher and lower levels of risk of AD is different and, if so, whether there is a relationship with the visual processing at the cortical level in the high-risk group, similar to the one found for macular disease. Second, similarly, to assess if there are differences in contrast sensitivity, and, if so, whether there is a relationship with the visual processing at the cortical level in the high-risk group, similar to the one found for macular disease. Finally, to assess whether there are differences in the thickness of any of the retinal layers and whether the thickness of these layers is related to visual processing in the high-risk group. Early findings in high-risk relatives of AD patients may provide a new perspective in the search for biomarkers for early diagnosis, decades before the age of onset of AD’s typical symptoms.

## Methods

### Participants

This investigation was a part of a project titled: “*Características cognitivas y neurofisiológicas de personas con alto riesgo para el desarrollo de demencia: una aproximación multidimensional*” (COGDEM), a prospective longitudinal study targeting the identification and progression of possible biomarkers capable of detecting subjects at higher risk of developing dementia in multiple domains [25]. The total sample of the study consisted of 251 cognitively healthy subjects, all of which signed the informed consent form. The research followed the tenets of the Declaration of Helsinki, and the San Carlos Clinical Hospital Ethics Committee approved the study with the internal code 18/422-E_BS.

As inclusion criteria, participants were required to have a complete ophthalmological evaluation and characterization of *APOE* alleles, have valid MEG recordings, and fulfill ophthalmological inclusion criteria (see below). Exclusion criteria were defined as scoring less than 25 on the Mini-Mental State Examination (MMSE) after adjusting their score to their age and educational level, following the procedure described in Blesa et al. [26] with a Spanish population. Participants who scored lower than 10% of their normative population in the Logical Memory subscale of the Wechsler Memory Scale III [27] in both units recall and themes recall scores, were not considered further. Other exclusion criteria were having a history of neurological or psychiatric disorders or any serious medical condition or showing brain abnormalities in magnetic resonance imaging (MRI). In total, 107 subjects were subsequently excluded according to these criteria, and the final sample consisted of 144 subjects.

**Fig. 1:**
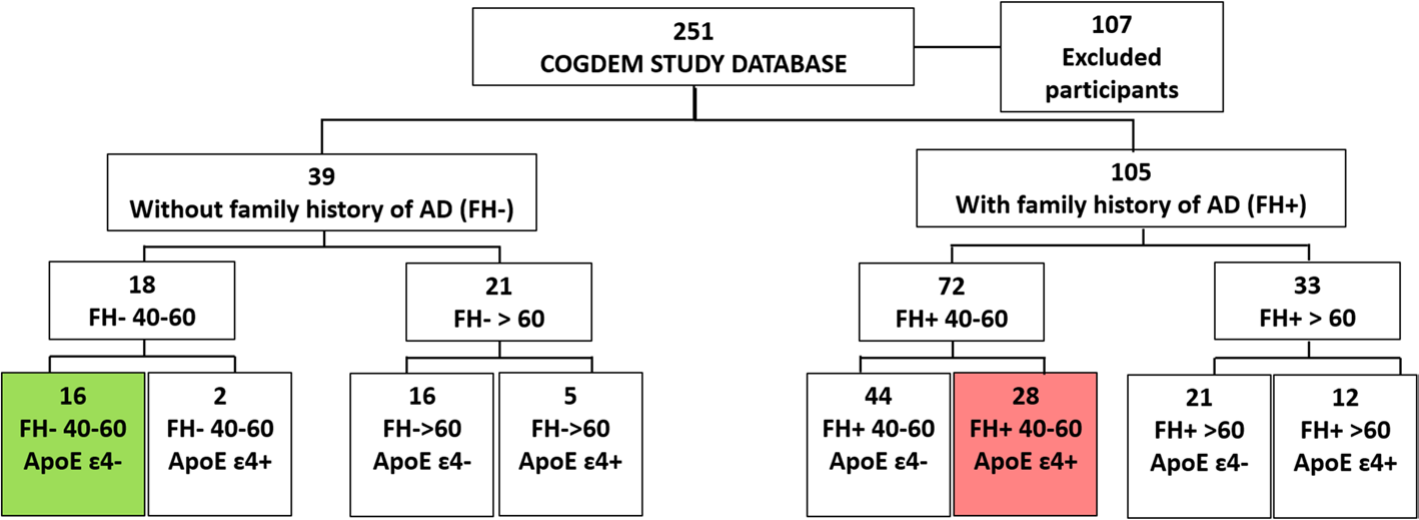
Flow diagram of study participants. The participants without family history of AD (FH −) and non-carriers of ApoE ɛ4 (ApoE ɛ4 −) in green and the participants with family history of AD (FH +) and carriers of ApoE ɛ4 (ApoE ɛ4 +) in red

The resulting sample was divided into two groups: participants who had at least one first grade relative with AD (FH +) and those who had no family history of the disease (FH −). Relatives’ AD diagnosis was verified after a medical history review by a multidisciplinary diagnostic consensus panel. Only those diagnoses made under international criteria or certified by autopsy reports of the relative were accepted. In addition, in order to avoid the influence of the variable “age,” which greatly affects visual function, especially contrast sensitivity [28–30], the sample was divided into a subsample ranging between 40 and 60 years of age, and a subsample over 60 years of age. Finally, each subsample was stratified according to allelic characterization for the *APOE* ɛ4 gene. The details of each group are shown in Fig. 1.

Due to limitations in sample size and statistical power, as well as asymmetries between the size of the groups and the demographics of some of them, the more natural analysis for the subgroups, a 2 × 2 × 2 design, taking into account age, family history, and APOE, was not feasible. Instead, visual functioning analyses were performed for the bigger groups (FH − vs. FH +) and the more restricted subgroups (FH − _40-60ɛ4−_ vs. FH + _40-60ɛ4+_), given the big influence of age over the variables. Additional comparisons between older groups were performed to assess if the patterns were distinct from the ones found in the younger group. Given the lack of differences among the older groups, further correlation analyses were not performed.

The analyses of retinal structure were performed only for the more restrictive subgroups because the analysis of the larger groups (FH − and FH +) has been published previously [13]. Furthermore, as changes in visual function appear between the subgroups (FH − _40-60ɛ4−_ vs. FH + _40-60ɛ4+_), it would be interesting to complement the study by OCT analysis in these subgroups.

When testing for possible demographic confounding variables, the only significant difference was found in the age of FH − and FH + . This difference disappears in the more restricted groups with ages between 40 and 60, which constitutes an additional reason to perform the analyses in this subgroup. The detailed data is shown in Table 1.

**Table 1:** Participant demographics

	FH − **(*n*=39)**	FH + **(*n*=105)**	Statistics **(*p*-value)**	Effect size	FH − _40-60ɛ4−_ **(*n*=16)**	FH + _40-60ɛ4+_ **(*n*=28)**	Statistics **(*p*-value)**	Effect size
Sex (M/F)	15/24	38/67	0.846	0.02 (0.00, 0.19)	3/13	9/19	0.490	0.14 (0.00, 0.42)
Age (y)	61.1 ± 7.6	57.7 ± 6.3	*0.029	0.18 (0.02, 0.35)	54.0 ± 2.5	53.6 ± 4.5	0.742	0.05 (0.00, 0.34)
Education	4.7 ± 0.6	4.6 ± 0.6	0.404	0.07 (0.00, 0.23)	4.7 ± 0.6	4.6 ± 0.6	0.539	0.09 (0.00, 0.36)
MMSE	28.3 ± 1.0	28.3 ± 1.1	0.750	0.03 (0.00, 0.20)	28.1 ± 0.9	28.6 ± 1.0	0.102	0.25 (0.02, 0.52)

Values are presented as mean ± SD. Effect size is presented as Cramer’s V (confidence interval) for sex and Wilcoxon effect size (confidence interval) for the other variables. Statistical analyses were performed using the chi-square test (sex) and Wilcoxon rank sum test with continuity correction (others). Education was measured as the maximum level of formal education received. MMSE was standardized according to Blesa [26]. *p< 0.05

### Genotyping

The *APOE* genotyping was carried out at the San Carlos Clinical Hospital in Madrid. DNA was extracted from whole blood in ethylenediamine tetra-acetic acid (EDTA), using standard DNA isolation methods (DNAzol®; Molecular Research Center, Inc., Cincinnati, OH, USA). *APOE* haplotype was determined by analyzing single-nucleotide polymorphisms (SNPs) rs7412 and rs429358 genotypes with TaqMan assays (C____904973_10 and C___3084793_20, respectively), using an Applied Biosystems 7500 Fast Real-Time PCR machine (Applied Biosystems, Foster City, CA).

*APOE ε*3/*ε*4 and *APOE ε*4/*ε*4 subjects were considered *APOE ε*4 + , while *APOE ε*2/*ε*3 and *APOE ε*3/*ε*3 subjects were considered *APOE ε*4 − .

### Ophthalmological analysis

As previously described in López-Cuenca et al. [13], all participants completed a telephone screening interview to determine the status of their visual health. Those who were confirmed to be free of any pathology were examined at the Ramon Castroviejo Institute of Ophthalmic Research clinic (Madrid, Spain). A complete ophthalmologic examination, including visual acuity, refraction, applanation tonometry (Perkins MKII tonometer), CSV-1000E test, and OCT examination, was performed, and only participants who had no ocular disease, a best-corrected visual acuity of 0.5 dec, a spherocylindrical refractive error of less than ± 5, and an intraocular pressure of less than 20 mmHg were included.

### Visual acuity

As previously described in Salobrar-García et al. [31], a standard clinical Snellen eye chart (decimal scale) was employed to determine the monocular best-corrected visual acuity. Visual acuity was measured with the subject’s subjective refraction. Patients started reading each row from the top towards the bottom of the chart and the test ended when the subjects were not able to recognize at least five out of eight letters (an approximation of 56.25%, the steepest point of the psychometric acuity function).

### Contrast sensitivity function

The contrast sensitivity test was performed to measure the contrast sensitivity function under the same conditions for all participants, as previously described in Salobrar-García et al. [31]. A detailed description of the procedure can be found in the Supplementary Materials.

### Optical coherence tomography

Macular thickness of each layer and peripapillary retinal nerve fiber layer (pRNFL) were measured using Spectralis OCT (Heidelberg Engineering, Heidelberg, Germany) as previously described in López-Cuenca et al. [13]. A detailed description of the procedure can be found in the Supplementary Materials.

The colorimetric representation of the changes in the macular and peripapillary thickness between the study groups was done with the Excel software and the color scale function. Areas where no difference can be found are colored in white, sectors that presented thinning among the FH + _40-60ɛ4+_ group and FH − _40-60ɛ4−_ are colored in blue tones, and those that showed thickening are colored in red tones. The color tone is provided directly by the software based on the thickness variation.

### Magnetic resonance imaging acquisition

For each subject, a T1-weighted anatomical brain MRI scan was acquired at the San Carlos Clinical Hospital in Madrid, with a General Electric 1.5 T magnetic resonance scanner, using a high-resolution antenna and a homogenization Phased array Uniformity Enhancement filter (Fast Spoiled Gradient Echo sequence, TR/TE/TI = 11.2/4.2/450 ms; flip angle of 12°; 1 mm slice thickness, 256 × 256 matrix, and FOV of 25 cm).

### Magnetoencephalography

#### Data acquisition

The electrophysiological activity of each participant was recorded at the *Centro de Tecnología Biomédica* during the performance of a cognitive task, described at length in Serrano et al. [32]. In brief, the task consisted of a delayed match-to-sample paradigm with faces as stimuli. For the present article, only the visual-related activity generated after the presentation of the faces will be addressed. The task comprised 128 trials, each containing two face presentations, resulting in a total of 256 face-locked events. All faces were neutral, Caucasian, adult male and female faces on a gray noise background and were kept on screen for 1 s in each presentation.

A detailed description of the task, MEG specifications, preprocessing performed, and source reconstruction methods can be found in the Supplementary Materials.

#### M100 latency

The visual evoked field (VEF) was generated using the face visualization task aforementioned. Each participant had, at least, 100 valid phase-locked events (211.48 ± 30.53; mean ± standard deviation). There were no statistically significant differences in the number of epochs between groups (*t* = 0.702; *p* = 0.487).

**Fig. 2:**
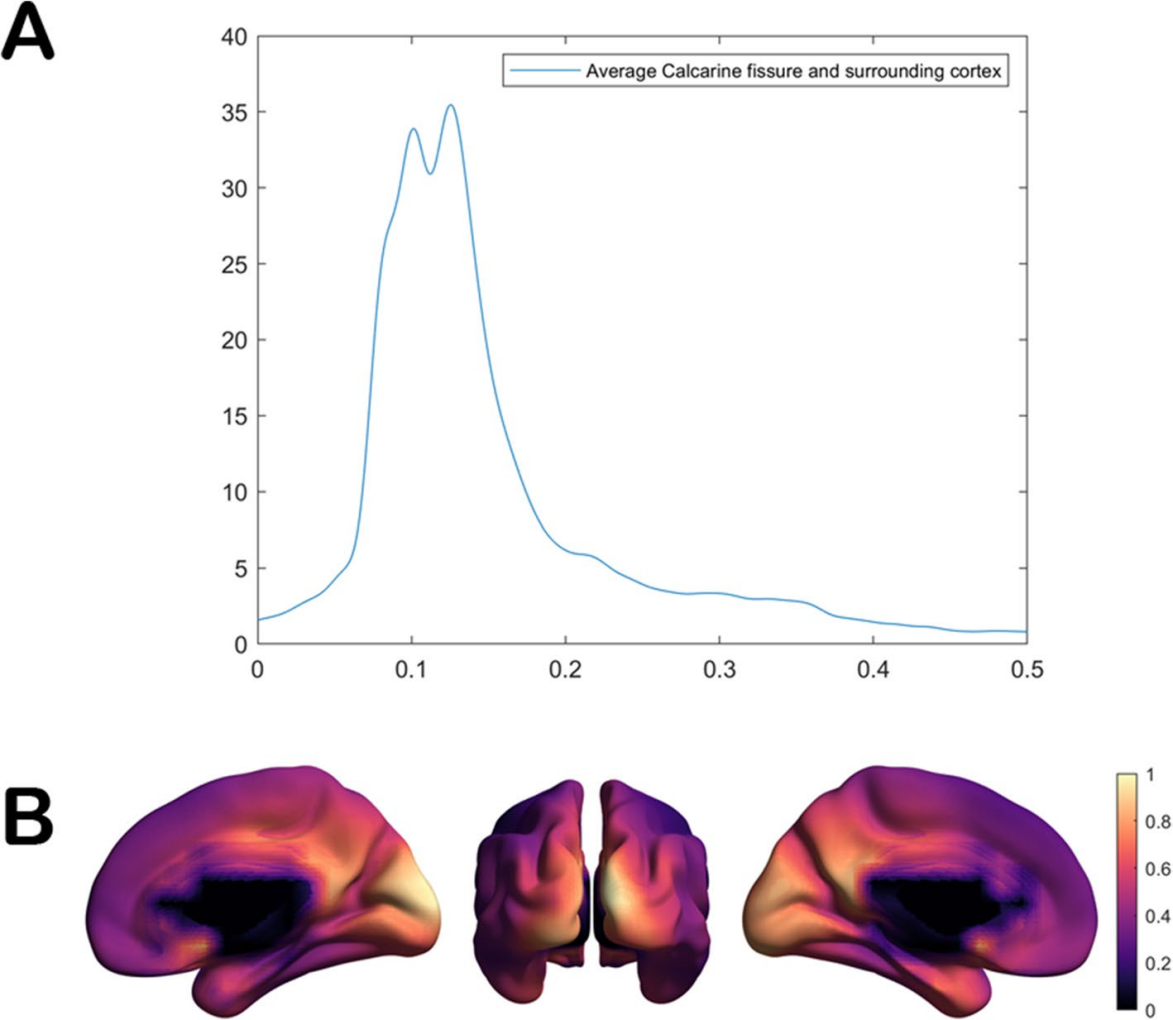
M100 latency. **A** Grand-averaged power at the calcarine fissure. **B** Grand-averaged power in the brain at 100 ms

For the present study, only the M100 component of the VEF was addressed. The individual latency values were defined as the point of maximal activation of the calcarine cortex, as defined by the automated anatomical labeling (AAL) atlas [33]. Figure 2A, B shows the grand-average of the activation in these areas for a subsample of 133 individuals, showing the average M100 power.

#### Time–frequency analysis

Evoked field amplitude in MEG is highly dependent on the participant’s position in the scan, resulting in an unreliable metric. Therefore, we analyze the level of brain activity evoked by the face presentation using a time–frequency (TF) analysis.

TF representation was calculated for a 1000-ms time window, from 500 ms before to 500 ms after the face presentation. Epochs were analyzed in the time–frequency domain using a 5-cycle Gaussian Morlet wavelet with 1 Hz steps from 2 to 30 Hz. In order to avoid edge effects, all epochs had 2 s of real data at each side as padding. Resulting data were corrected by the average basal activity before the presentation of the stimulus, resulting in a relative change representation.

**Fig. 3:**
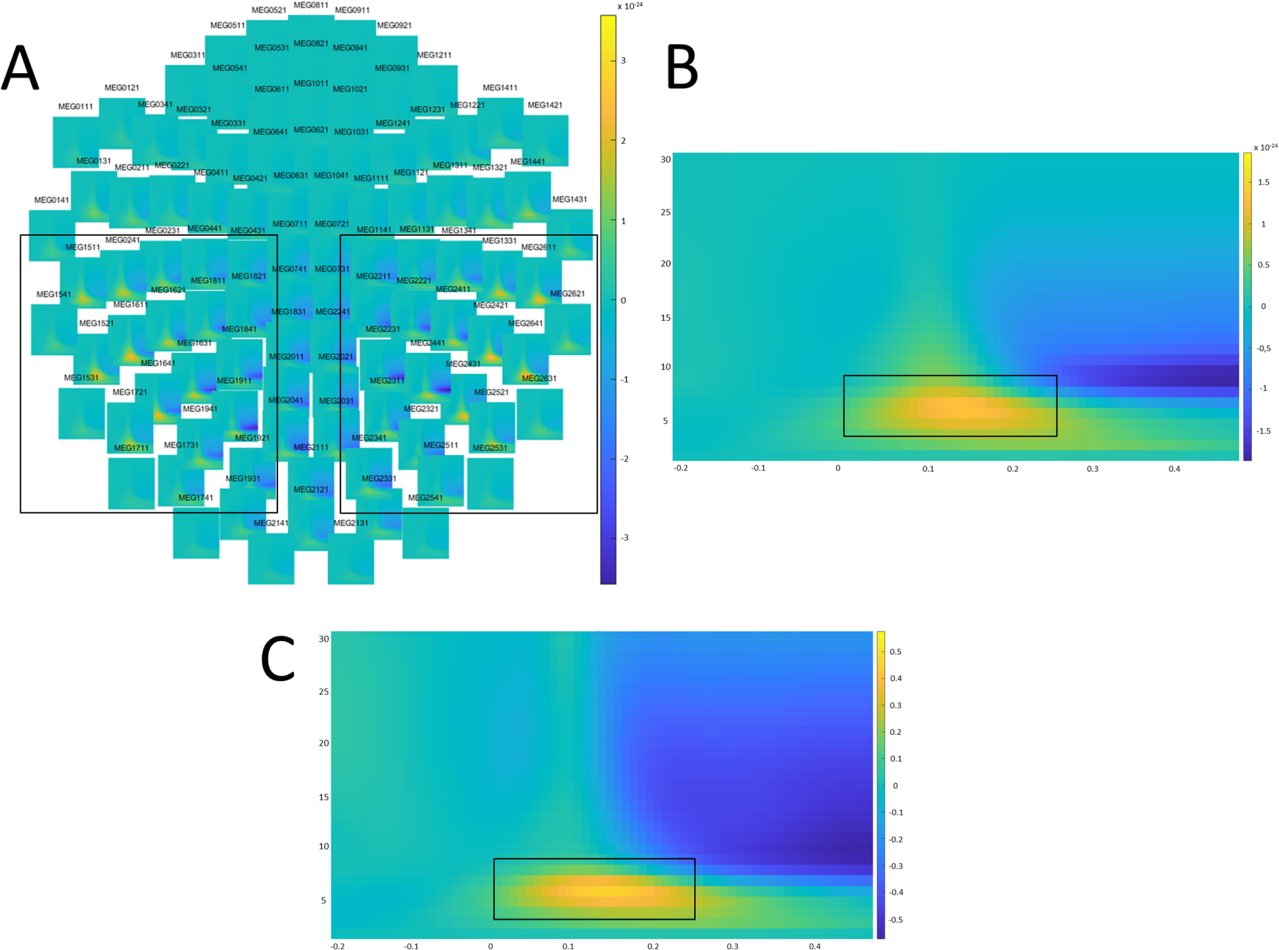
Visual response related time–frequency analysis.** A** Grand-averaged TF representation in the sensor space. **B** Grand-averaged TF representation in the sensors inside the **A** rectangles. **C** Grand-averaged TF representation in the source space (calcarine cortex). Rectangles in **A** indicate the sensors in which the activity is best perceived. Rectangles in **B** and **C** indicate the TF range in which the activity is more prevalent, which is later used for the analysis

The subsequent analyses were restricted to the range in which the visual response takes place. To determine this range, a grand-average TF response for 133 participants (see Fig. 3A–C) was calculated. According to this response, the analyses were performed in the range of frequencies between 4 and 10 Hz, and the range of latencies between 0 and 250 ms.

#### Statistical analysis

Chi-squared test was used to perform comparisons between groups in qualitative variables.

**Fig. 4:**
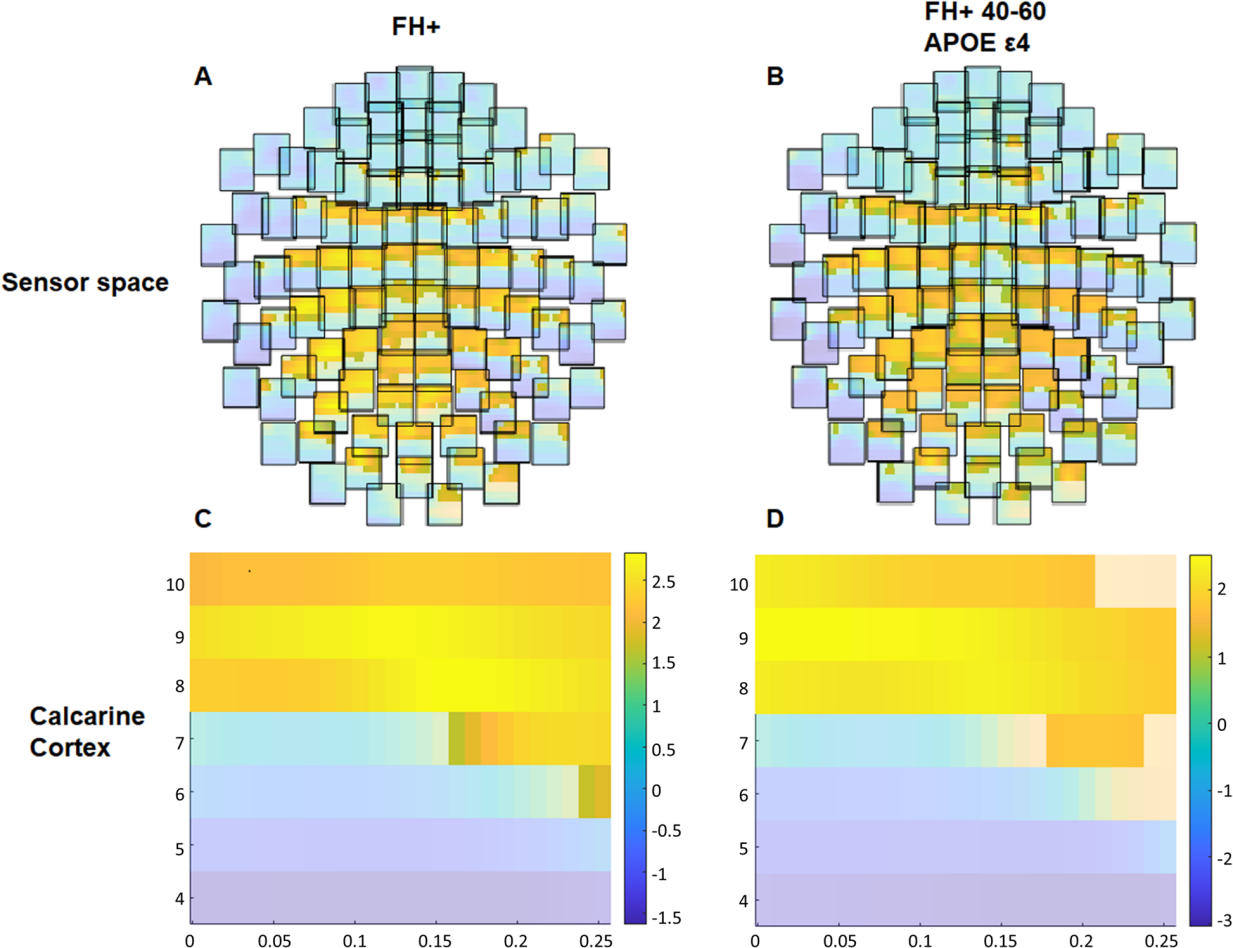
Cluster-based permutation test for the relationships of TF activity with visual function. Blue colors indicate negative correlations, while yellow colors indicate positive correlations. The time–frequency-sensor triplets pertaining to the significant cluster are shown in solid colors. **A** Correlation between visual acuity and TF in the FH + group (sensors); **B** correlation between visual acuity and TF in the FH + _40-60ɛ4+_ group (sensors); **C** correlation between visual acuity and TF in the FH + group (calcarine); **D** correlation between visual acuity and TF in the FH + _40-60ɛ4+_ group (calcarine)

Wilcoxon rank sum test with continuity correction was used to perform comparisons between groups in continuous variables, and Spearman correlation coefficients were obtained to assess the relationship between ophthalmological and electrophysiological variables. These methods were chosen as they make less assumptions regarding the data distribution. Additionally, Spearman correlation is capable of assessing non-linear monotonic relationships.

The correlation differences between the groups were calculated transforming the correlations to Fisher *Z* scores.

To measure effect sizes, Cramer’s V and its 95% confidence interval were calculated for the comparisons between groups in qualitative variables. For the comparisons between groups in continuous variables, effect size calculations from the Mann–Whitney *U* test and its 95% confidence interval, which has an interpretation analogous to a correlation coefficient, were estimated [34]. For correlations, the Spearman correlation coefficient acts as the size effect statistic.

To evaluate the TF power correlation with visual acuity, contrast sensitivity, and macular thickness, two approaches were taken. First, the analyses were performed in the sensor space, using cluster-based permutation test (CBPT) for multiple comparison corrections, with a Monte Carlo procedure, implementing 10,000 randomizations and a significance threshold of 0.05, taking sensors, time, and frequency as dimensions. Second, the analyses were also performed in the source space, averaging the TF sources belonging to the calcarine cortex as defined by the AAL atlas, and using CBPT with the same parameters, taking time and frequency as dimensions. As previously noted, both types of analysis were restricted to frequencies between 4 and 10 Hz and latencies between 0 and 250 ms.

We used false discovery rate (FDR) to correct for multiple comparisons. Within each research question, we applied FDR three times, once to correct the differences between groups, and once to correct the relationship between the ophthalmological measure and the M100 latency and the TF power for each group.

The preprocessing of MEG data and the CBPT analyses were performed in MATLAB R2019b (The Mathworks, Inc., Natick, MA), using the Fieldtrip package blindly to the group each subject belonged to. All other analyses were performed using R 3.6.2.

## Results

To address the first and second research questions, we have depicted the detailed results for the comparisons between groups in visual acuity and contrast sensitivity in different spatial frequencies in Table 2, and the correlations between these variables and the electrophysiological measures in Tables 3 and 4 and Fig. 4.

**Table 2:** Visual function

	FH − $(n=39)$	FH + $(n=105)$	Statistics (*p*-value)	Effect size	FH − _40-60ɛ4−_ $(n=16)$	FH + _40-60ɛ4+_$(n=28)$	Statistics (*p*-value)	Effect size
VA	0.990 ± 0.094	1.030 ± 0.096	*0.043	0.17 (0.02, 0.32)	0.981 ± 0.040	1.036 ± 0.095	*0.018	0.36 (0.14, 0.54)
CS-3	1.752 ± 0.121	1.741 ± 0.142	0.660	0.04 (0.00, 0.21)	1.724 ± 0.121	1.749 ± 0.148	0.560	0.09 (0.00, 0.37)
CS-6	1.971 ± 0.182	1.990 ± 0.172	0.597	0.05 (0.00, 0.22)	1.972 ± 0.171	2.029 ± 0.213	0.311	0.15 (0.00,0.43)
CS-12	1.592 ± 0.177	1.675 ± 0.177	0.080	0.15 (0.00, 0.32)	1.544 ± 0.169	1.675 ± 0.182	*0.025	0.34 (0.06, 0.56)
CS-18	1.164 ± 0.160	1.170 ± 0.197	0.833	0.02 (0.00, 0.19)	1.144 ± 0.164	1.218 ± 0.197	0.125	0.23 (0.00, 0.50)
	FH − > 60 $(n=21)$	FH + > 60 $(n=33)$	Statistics (*p*-value)	Effect size	FH − > 60ɛ4 − $(n=16)$	FH + > 60ɛ4 + $(n=12)$	Statistics (*p*-value)	Effect size
VA	1.005 ± 0.120	0.991 ± 0.068	0.192	0.18 (0.00, 0.41)	1.006 ± 0.139	1.000 ± 0.074	0.540	0.12 (0.00, 0.45)
CS-3	1.812 ± 0.162	1.694 ± 0.131	*0.011	0.36 (0.11, 0.60)	1.824 ± 0.179	1.686 ± 0.166	0.068	0.37 (0.05, 0.70)
CS-6	1.968 ± 0.199	1.953 ± 0.153	0.709	0.05 (0.00, 0.34)	1.949 ± 0.220	1.977 ± 0.169	0.779	0.06 (0.00,0.45)
CS-12	1.644 ± 0.178	1.629 ± 0.178	0.984	0.00 (0.00,0.31)	1.659 ± 0.186	1.597 ± 0.189	0.591	0.11 (0.00, 0.49)
CS-18	1.203 ± 0.150	1.170 ± 0.227	0.366	0.13 (0.00, 0.33)	1.186 ± 0.126	1.158 ± 0.296	0.537	0.13 (0.00, 0.58)

Values are presented as mean ± SD. Effect size is presented as *r* (*r* confidence interval). Statistical analyses were performed using Wilcoxon rank sum test with continuity correction. *VA* visual acuity, *CS* contrast sensitivity. Numbers after CS: spatial frequency in cpd. *p<0.05

**Table 3:** M100 peak latency relationship with visual acuity, contrast sensitivity, and macular thickness

	FH −			FH +			FH − _40-60ɛ4−_			FH + _40-60ɛ4+_		
	*n*	rho	*p*	*n*	rho	*p*	*n*	rho	*p*	*n*	rho	*p*
VA	32	0.033	0.571	94	− 0.180	*0.041	14	0.066	0.588	26	− 0.408	*0.019
CS-3	30	− 0.021	0.456	90	− 0.028	0.398	14	− 0.472	*0.044	26	− 0.204	0.159
CS-6	30	− 0.026	0.470	90	− 0.008	0.470	14	0.078	0.604	26	− 0.066	0.375
CS-12	30	0.247	0.906	90	0.084	0.785	14	0.061	0.582	26	− 0.132	0.260
CS-18	30	0.085	0.672	90	− 0.007	0.473	14	− 0.194	0.253	26	0.050	0.596
IPL-N1							13	0.104	0.735	20	− 0.029	0.903
IPL-N2							13	0.048	0.877	20	− 0.062	0.796
IPL-S1							13	− 0.184	0.548	20	0.063	0.791
IPL-I1							13	− 0.096	0.754	20	− 0.182	0.443
IPL-I2							13	− 0.044	0.886	20	− 0.475	*0.034
INL-I2							13	− 0.152	0.621	19	− 0.370	0.119

Spearman correlations with visual acuity and contrast sensitivity were left tailed contrasts, due to the theoretical hypotheses, while those with macular thickness were two-tailed. The differences in *n* with the original groups were due to invalid results in MEG or MRI. *p<0.05

**Table 4:** Cluster-based permutation test for the relationships of TF activity with visual function and retinal thickness

	FH −			FH +			FH − _40-60ɛ4−_			FH + _40-60ɛ4+_		
	*n*	rho	*p*	*n*	rho	*p*	*n*	rho	*p*	*n*	rho	*p*
Sensor space												
VA	32	0.377	0.502	95	0.234	*0.005	14	0.488	0.604	27	0.450	*0.008
CS-3	30	0.382	0.550	91	0.202	0.469	14	0.596	0.118	27	0.360	0.095
CS-6	30	0.365	0.741	91	0.195	0.478	14	0.573	0.396	27	0.366	0.371
CS-12	30	0.337	0.893	91	0.191	0.435	14	0.568	0.840	27	0.361	0.092
CS-18	30	0.363	0.3321	91	0.215	0.205	14	0.486	0.679	27	0.476	*0.036
IPL-N1							13	0.624 − 0.653	1 1	21	0.514 − 0.480	1 1
IPL-N2							13	0.630 − 0.581	0.852 1	21	− 0.492	0.267
IPL-S1							13	0.616 − 0.641	1 1	21	0.500	0.351
IPL-I1							13	0.633 − 0.601	0.995 1	21	0.498 − 0.503	0.695 1
IPL-I2							13	− 0.647	1	21	− 0.487	0.203
INL-I2							13	0.636 − 0.602	0.428 1	20	− 0.542	1
Source space (Calcarine cortex)												
VA	32	-	-	94	0.246	0.007	14	-	-	26	0.399	0.034
CS-3	30	-	-	90	-	-	14	0.470	0. 256	26	0.355	0.219
CS-6	30	-	-	90	-	-	14	0.527	0.200	26	0.336	0.290
CS-12	30	-	-	90	-	-	14	-	-	26	-	-
CS-18	30	0.331	0.202	90	-	-	14	-	-	26	0.436	0.028
IPL-N1							13	-	-	21	-	-
IPL-N2							13	-	-	21	-	-
IPL-S1							13	-	-	21	-	-
IPL-I1							13	-	-	21	− 0.458	0.183
IPL-I2							13	-	-	20	− 0.530	0.332
INL-I2							13	-	-	21	-	-

At the top, results in the sensor space. At the bottom, results in the source space, averaged in calcarine cortex following AAL atlas. Correlations with visual acuity and contrast sensitivity were left tailed contrasts, due to the theoretical hypotheses, while those with macular thickness were two-tailed. The differences in *n* with the original groups were due to invalid results in MEG or MRI. *p<0.05

**Fig. 5:**
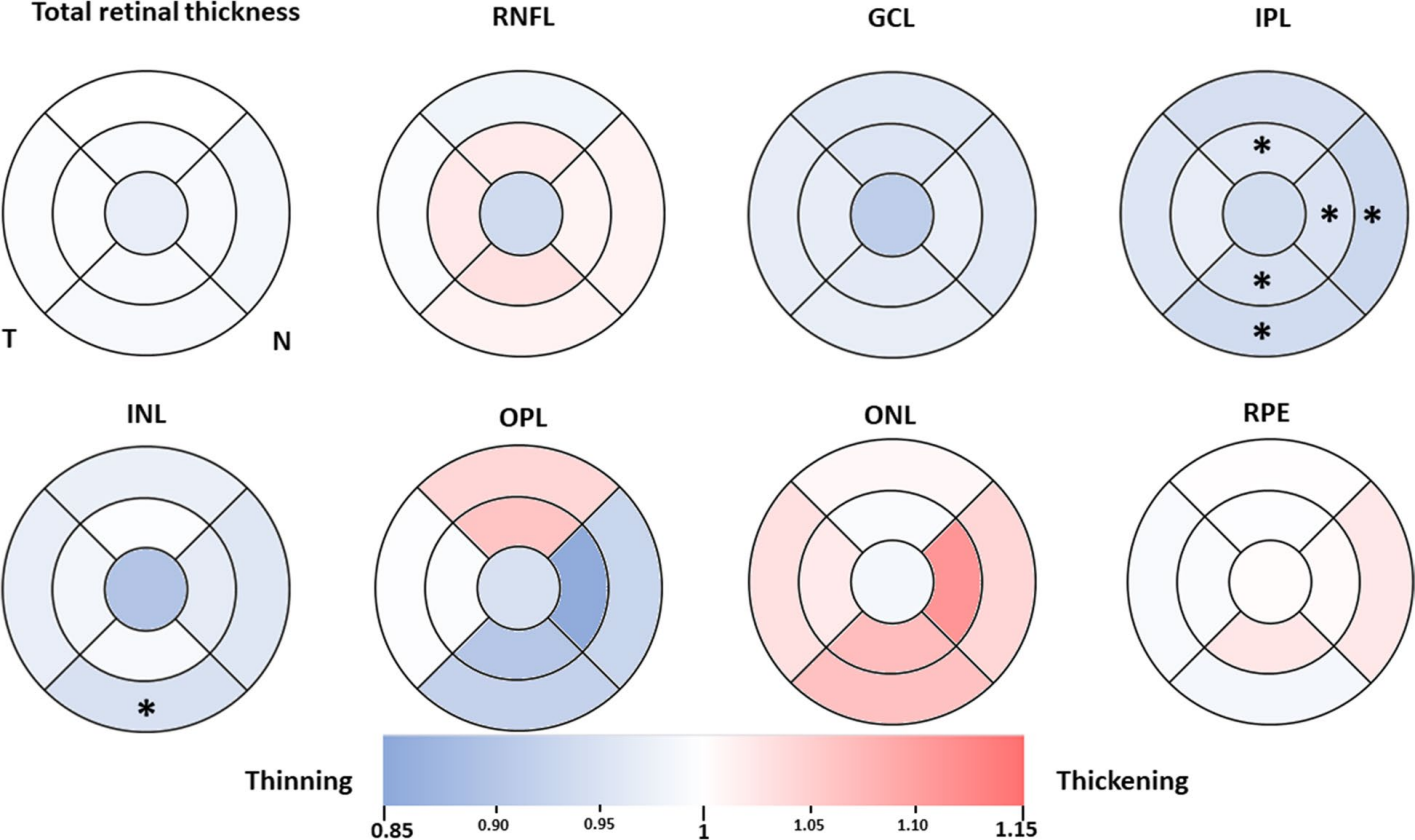
Colorimetric differences in the retinal thickness in each layer between the groups. FH + _40-60ɛ4+_ vs FH − _40-60ɛ4−_ in the macular OCT rings. In red, thickening; in blue, thinning. (RNFL: retinal nerve fiber layer; GCL: ganglion cell layer; IPL: inner plexiform layer; INL: inner nuclear layer; OPL: outer plexiform layer; ONL: outer nuclear layer; RPE: retinal pigment epithelium). * *p* < 0.05. Wilcoxon rank sum test with continuity correction. The significance is lost after correction for multiple comparisons

To address the third research question, some of the OCT images were not of sufficient quality to extract the measurements. Consequently, those subjects were not included in the statistical analyses and the final group sizes were 15 FH − _40-60ɛ4−_ and 22 FH + _40-60ɛ4+_.

When performing main comparisons with magnetoencephalography measures, no significant differences were found in the M100 peak latency, neither when comparing the less strict groups (FH − : 0.152 ± 0.019, FH + : 0.153 ± 0.021, W = 1499.50, *p* = 0.982, *r* < 0.01 [0.00, 0.19]), nor when comparing the more restricted ones (FH − _40-60ɛ4−_: 0.155 ± 0.022, FH + _40-60ɛ4+_: 0.152 ± 0.023, W = 193.00, *p* = 0.765, *r* = 0.05 [0.00, 0.37]). When performing main comparisons in TF activity, no significant clusters were found, neither between FH − and FH + , nor between the more restricted FH + _40-60ɛ4+_ and FH − _40-60ɛ4−_ subgroups. To make sure that the latency and the TF response were independent measures, a supplementary analysis was performed to assess the correlation between them. No significant results were observed, neither in the sensor space nor in the calcarine cortex.

### Visual acuity

FH + and FH + _40-60ɛ4+_ show higher visual acuity than FH − and FH − _40-60ɛ4−_ respectively. However, no significant differences were found in the older sample (Table 2).

Additionally, a negative relationship between visual acuity and M100 latency was found among FH + and FH + _40-60ɛ4+_ (Table 3). Finally, the TF analyses in the sensor space and in the source space (calcarine cortex) showed that the FH + and FH + _40-60ɛ4+_ groups presented positive correlation clusters between the TF response and visual acuity in both analyses (Table 4). All differences and correlations remained significant after correction for multiple comparisons.

No significant correlations were found between visual acuity and either electrophysiological measure in the low-risk groups (Tables 3 and 4).

The differences between the correlations of the FH − and FH + groups between VA and the M100 latency did not reach significance (*Z* = 0.866, *p* = 0.193) nor the ones between the more restricted FH + _40-60ɛ4+_ and FH − _40-60ɛ4−_ groups (*Z* = 1.362, *p* = 0.087). However, the second comparison shows a tendency close to the significance threshold.

### Contrast sensitivity

Subjects between 40 and 60 years of age at greater risk tend to show higher CS values. However, after correcting for multiple comparisons, the only previously significant results among FH + _40-60ɛ4+_ (greater CS at 12 cycles per degree; cpd) and FH −  > 60 (greater CS at 3 cpd) (Table 2) were no longer significant.

When addressing the correlation between CS and the M100 latency, the FH − _40-60ɛ4−_ group presented a negative relationship between latency and contrast sensitivity in 3 cpd (see Table 3). Likewise, the FH + _40-60ɛ4+_ group showed a positive correlation between the TF response and contrast sensitivity in 18 cpd (see Table 4) both in the sensor and source space. However, these differences did not survive correction for multiple comparisons.

### Optical coherence tomography

The differences in macular thickness between FH + _40-60ɛ4+_ and FH − _40-60ɛ4−_ were significant in the IPL and the INL only. However, they did not survive the correction for multiple comparisons. No significant differences between the groups were found in the total retinal thickness or any sector of the macular RNFL, GCL, OPL, ONL, or RPE Fig. 5.

Regarding the IPL, the FH + _40-60ɛ4+_ group presented decreases in thickness in comparison to the FH − _40-60ɛ4−_ in (i) the nasal sector, both in the inner (N1: 41.82 ± 2.59 vs. 43.93 ± 2.49, mean ± standard deviation; W = 239.50, *p* = 0.021; Cohen’s *d* =  − 0.83; [− 1.54 − 0.12], confidence interval at 95% certainty) and outer (N2: 30.00 ± 2.47 vs. 32.27 ± 2.89; W = 237.00, *p* = 0.025; Cohen’s *d* =  − 0.86 [− 1.57 − 0.15]) rings, (ii) the superior sector, in the inner (S1: 40.91 ± 2.20 vs. 42.73 ± 1.98; W = 247.00, *p* = 0.010; Cohen’s *d* =  − 0.86 [− 1.57 − 0.15]) ring, and (iii) the inferior sector, both in the inner (I1: 40.32 ± 2.51 vs. 42.60 ± 2.77; W = 250.00, *p* = 0.008; Cohen’s *d* =  − 0.87 [− 1.58 − 0.16]) and outer (I2: 26.95 ± 2.26 vs. 28.93 ± 1.94; W = 250.50, *p* = 0.008; Cohen’s *d* =  − 0.93 [− 1.64 − 0.21]) rings.

Regarding the INL, the FH + _40-60ɛ4+_ group presented a statistically significant decrease in thickness in comparison to the FH − _40-60ɛ4−_ in the outer ring of the inferior sector only (*I*^2^ 30.81 ± 2.29 vs. 33.00 ± 2.36; W = 234.00, *p* = 0.014; Cohen’s *d* =  − 0.94 [− 1.67 − 0.22]). However, as stated before, all of these results were not significant after correction for multiple comparisons.

FH + _40-60ɛ4+_ presented a negative relationship between the M100 latency and the retinal thickness of the IPL-I2, although it did not reach significance after correction for multiple comparisons.

In the pRNFL, no statistically significant differences in thickness were found when comparing the FH + _40-60ɛ4+_ group to the FH − _40-60ɛ4−_ group. Results are shown in supplementary Fig. 2.

## Discussion

A variety of visual impairments have been previously associated with neurological diseases. Regarding AD, our group has found that patients show a decrease in visual acuity and contrast sensitivity in the early clinical stages [31] and that these alterations also exist in intermediate stages of the disease [8]. Different authors have also reported a loss of visual acuity in AD patients [35–37], and even visual hallucinations when the impairment is severe [38, 39]. Other authors, however, do not find significant differences [40, 41].

Regarding our first research question, surprisingly, we found an early increase in visual acuity associated with a high risk of future dementia, both when comparing relatives versus non-relatives, and when comparing narrower and smaller subgroups within a limited age range and maximizing risk differences. The fact that this difference is found under both classification systems suggests that this is a stable indicator of visual function status in subjects at risk of AD, who are still cognitively unimpaired. Optic nerve and retinal degeneration is well documented in AD [42, 43]. It has been shown that the P100 latency is delayed and has a smaller amplitude in optic nerve and macular diseases [24]. Moreover, previous studies have shown that there is a relationship between a greater visual acuity with a lower P100 latency and greater P100 amplitude in patients with macular disease [24]. We found similar results in the high-risk group that exhibits a relationship where higher visual acuity is associated with lower M100 latency and increased TF power. In contrast, no such relationship was found in the low-risk group. Taking both results together, this finding among high-risk subjects could constitute an indicator of early abnormalities in the biological substrates that affect both visual acuity and early visual potentials. Consequently, these measures could constitute not just a proxy to the state of the visual system, but also allow clinicians to track the disease progression. Given the known pattern of changes across the different stages of the disease, together with the ophthalmological measures, the electrophysiological pattern could represent an early, accessible, and non-invasive biomarker to track the pathology. Future studies could assess if the electrophysiological findings are also found using EEG, which is more readily available and accessible.

The increased visual acuity in participants at risk for AD seems at odds with previous findings pointing towards a progressive worsening of visual functioning in later clinical stages. We propose two possible explanations for this apparent discrepancy. On the one hand, the increased visual function could be a manifestation of an early compensatory response. On the other hand, the increase in visual function could be a manifestation of the hyperexcitability of retinal cells due to Aβ accumulation. It is well known that Aβ accumulation affects the normal functioning of inhibitory neurons in the brain [44], and recent evidence suggests that this may be mediated by an alteration in the dopaminergic system [45]. In Parkinson’s disease, where the dopaminergic system is impaired, a loss of dopaminergic cells and their synaptic contact with amacrine cells is observed [46]. It is possible that the modulatory dopaminergic activity in the retina of individuals at risk of developing AD is similarly affected by Aβ deposition, thus leading to an early hyperexcitability and increased visual acuity.

Decreased contrast sensitivity in mild cognitive impairment (MCI) or subjective cognitive decline (SCD) has been associated with p-tau and Aβ deposition in parietal, temporal, and especially occipital brain regions [47].Studies in patients with MCI have reported a significant general decrease of contrast sensitivity in all frequencies across the visual field, when measured by frequency doubling technology [47–49].

Regarding our second research question, high-risk subjects between 40 and 60 years of age presented a tendency towards higher CS values, especially at higher frequencies. However, after correction for multiple comparisons, no significant results remained. This could be due to the relatively small sample size or a lack of statistical power, and further research is needed to assess whether these trends are reliable and stable. Interestingly, although it was not part of the original research question, subjects over 60 years of age showed an opposite trend to that of young subjects, with lower CS values for the high-risk groups, especially at lower frequencies. This would support our original idea based on the literature that the older sample would behave differently on these measures, and validates the decision to perform the analyses separately for the older and younger groups.

Different authors have reported reduced m-RNFL thickness in AD and MCI patients, associated with smaller hippocampal volumes and worse cognitive scores [50]. A delay in the latency time of the rod-cone response in the retina of both MCI and AD patients has also been found [50]. The presence of APOE ɛ4 in mice also induces both structural and functional retinal impairments [51]. In previous studies of this research group, a reduction in the thickness of the IPL and INL of AD relatives who were carriers of the APOE ɛ4 allele was found when compared to their non-carriers with no family history counterparts [13].

Regarding our third research question, a similar thinning trend has been found in some sectors of the IPL and INL in the younger subgroup. These results, although only a trend that disappears after corrections for multiple comparisons, we believe are relevant and may constitute an interesting exploratory result, allowing subsequent studies.

If confirmed in subsequent studies, an alteration in the IPL would make biological sense based on previous literature, as participants at high risk have been found to show a decrease in the cholinergic activity of this layer, related to the accumulation of Aβ deposits [52, 53]. On the other hand, studies have shown substantial decreases in synaptic density (25–35%) 2 to 4 years before the onset of AD cognitive symptoms, associations between Aβ concentration in the brain and the degree of synaptic loss [54] and accumulation of hyperphosphorylated tau in subjects already diagnosed with AD, or presenting MCI [55]. Regardless of the underlying mechanisms, neuronal loss and atrophy, and synaptic changes have been consistently reported, suggesting retinal neuronal susceptibility in AD, analogous to that of the brain, making the study of structural changes a relevant one [56].

Additionally, although not part of our research questions, we would like to address the apparent inconsistency coming from the lack of differences in MEG measures between groups, in conjunction with the different patterns of correlations between these measures and visual acuity in the high-risk and low-risk groups.

There are inconsistencies in the literature regarding the existence of changes in the latency and amplitude of the P100 wave (EEG; analog of the M100 studied here) among AD patients. Many studies, especially those not specifically designed to measure visual function (i.e., cognitive functioning), do not find alterations in the P100 latency and amplitude [57]. Other studies, mainly those specifically designed to measure visual evoked potentials (VEPs), report a delay in P100 latency, as well as a decrease in its amplitude in AD patients [58–60]. Clearly, consistent measurement of differences in the VEPs in AD patients is not an easy task. Consequently, it does not seem surprising that we do not find differences in the latency of the M100 or the time–frequency power between cognitively healthy subjects who only differ in their risk for developing the disease.

Some of the main limitations of this study are the differences in age found between the FH + and FH − groups and the sample size of the restricted subgroups. Additionally, the MEG task was not originally designed to measure visual functioning, but cognition, so the visual stimuli and the number of trials were not designed with this purpose in mind, and could lack some power and specificity to measure small changes in primary visual activity. Also, the cross-sectional nature of this study makes it impossible to elucidate the temporal progression along the continuum of the patterns of the differences described here. Finally, we do not have biological biomarkers of the disease, which could support the results obtained.

Despite these limitations, the subgroups were strictly selected to minimize possible confounding factors and maximize the risk differences. Moreover, the extreme risk group, even though small, is over-represented in our sample, as it constitutes a small fraction of the general population. Additionally, being able to detect significant correlations between visual functioning and M100/TF in spite of the abovementioned limitations supports the robustness of our findings.

Finally, a key strength of this study is that this cohort is part of a longitudinal study. This will allow to track the pattern of changes, evaluate the evolution of the trends of structural and functional change, and elucidate the meaning of these initial findings that could constitute the initial physiological alterations associated with the beginning of the progression of the disease. In addition, including the study of other biomarkers of disease (such as plasma, saliva, or microbiota samples) could strengthen the conclusions of future studies.

## Conclusions

The present study assesses, for the first time, the relationship between ophthalmological and electrophysiological measures in healthy subjects at high risk of developing AD. Furthermore, this study offers a novel approximation to the field of AD biomarker identification. An increase in visual acuity is found among high-risk subjects compared to low-risk subjects. The high-risk groups also present a significant relationship where a higher visual acuity associates with a lower M100 latency and greater TF power measured with MEG, a relationship that was absent in the low-risk group. These findings constitute the first evidence of the consequences of early alterations of the visual processing at the functional and physiological level. These markers, which have not been previously explored, are easy to obtain, both in the retina and the primary visual cortex, and could constitute a new generation of biomarkers for the early detection of the disease and to evaluate the effect of potential pharmacological and non-pharmacological interventions.

## Supplementary Information

**Additional file 1: Supplementary Figure 1.** OCT Retinal representation of the layers. **Supplementary Figure 2.** Colorimetric representation of the peripapillary retinal nerve fiber layer thickness. **Supplementary Figure 3.** Schematic overview of the WM task. **Supplementary Methods detailed**

## Data Availability

The datasets during and/or analyzed during the current study available from the corresponding author on reasonable request.

## Abbreviations

AAL: Automated anatomical labeling
Aβ: Amyloid-beta
APOE: Apolipoprotein E gene
APOE ɛ4 +: Carrier of the ɛ4 allele of the Apolipoprotein E gene
APOE ɛ4 −: Non carrier of the ɛ4 allele of the Apolipoprotein E gene
CBPT: Cluster-based permutation test
cpd: Cycles per degree
CS: Contrast sensitivity
FH +: Family history of Alzheimer’s disease
FH −: No family history of Alzheimer’s disease
GCL: Ganglion cell layer
INL: Inner nuclear layer
IPL: Inner plexiform layer
MCI: Mild cognitive impairment
MEG: Magnetoencephalography
MMSE: Mini-Mental State Examination
MRI: Magnetic resonance imaging
OCT: Optical coherence tomography
ONL: Outer nuclear layer
pRNFL: Peripapillary retinal nerve fiber layer
RNFL: Retinal nerve fiber layer
RPE: Retinal pigment epithelium
SCD: Subjective cognitive decline
TF: Time frequency
VA: Visual acuity
VEF: Visual evoked field
VEP: Visual evoked potential
