# Supplementary material for: Early visual alterations in individuals at-risk of Alzheimer’s disease: a multidisciplinary approach
Source: Alzheimers Res Ther. 2023 Jan 24;15:19. doi: 10.1186/s13195-023-01166-0 (PMC9872347; doi:10.1186/s13195-023-01166-0)
Supplement: Supplementary file 1 — Additional file 1: Supplementary Figure 1. OCT Retinal representation of the layers. Supplementary Figure 2. Colorimetric representation of the peripapillary retinal nerve fiber layer thickness. Supplementary Figure 3. Schematic overview of the WM task. Supplementary Methods detailed [file 13195_2023_1166_MOESM1_ESM.docx]

**Supplementary Methods detailed**

**Contrast sensitivity function**

The CSV-1000E system (VectorVision, Greenville, OH, USA) with the best subjective visual acuity correction for far vision was used. This test monitors and self-calibrates the light level at 85cd/m2 by providing a fluorescent luminance source that allows backlighting of a translucent chart. This CSV-1000E system was performed at 98.5 inches per manufacturer's recommendation. The translucent chart presents four spatial frequencies: 3, 6, 12 and 18 cyc/deg and each was presented in a separate row of the test. Each row presented 17 circular patches 1.5 inches in diameter. The patches in each row had a grid ranging from very high contrast (1st sample patch) at the left end of the row decreasing in contrast along the row (16 remaining patches). In each column, one patch corresponds to a grid, while the other patch remained blank. The patient was asked to look at the first patch of the sample and look for the grating pattern in each column. While reading the row, the patient was to indicate whether the grating was visible in the top or bottom patch of each column. If the grating was not visible in either patch, the patient was asked to indicate that both patches were blank. An attempt was made to encourage the patient to guess whether a grid was at least partially visible as it approached the threshold. However, the patient was cautioned that if he or she was not able to see either grating, the response should be "both blank." The contrast level of the last correct response was recorded as threshold.

**Optical coherence tomography**

In the macular area, centered on the Glaucoma Module Premium APS, 121 dense B-scans were performed per eye. At the optic nerve head (ONH), a high-resolution 4-line radial scan and three-circle scans, both centred at the Bruch’s membrane opening (BMO), were acquired to provide highly reproducible retinal nerve fiber layer (RNFL) thickness results.


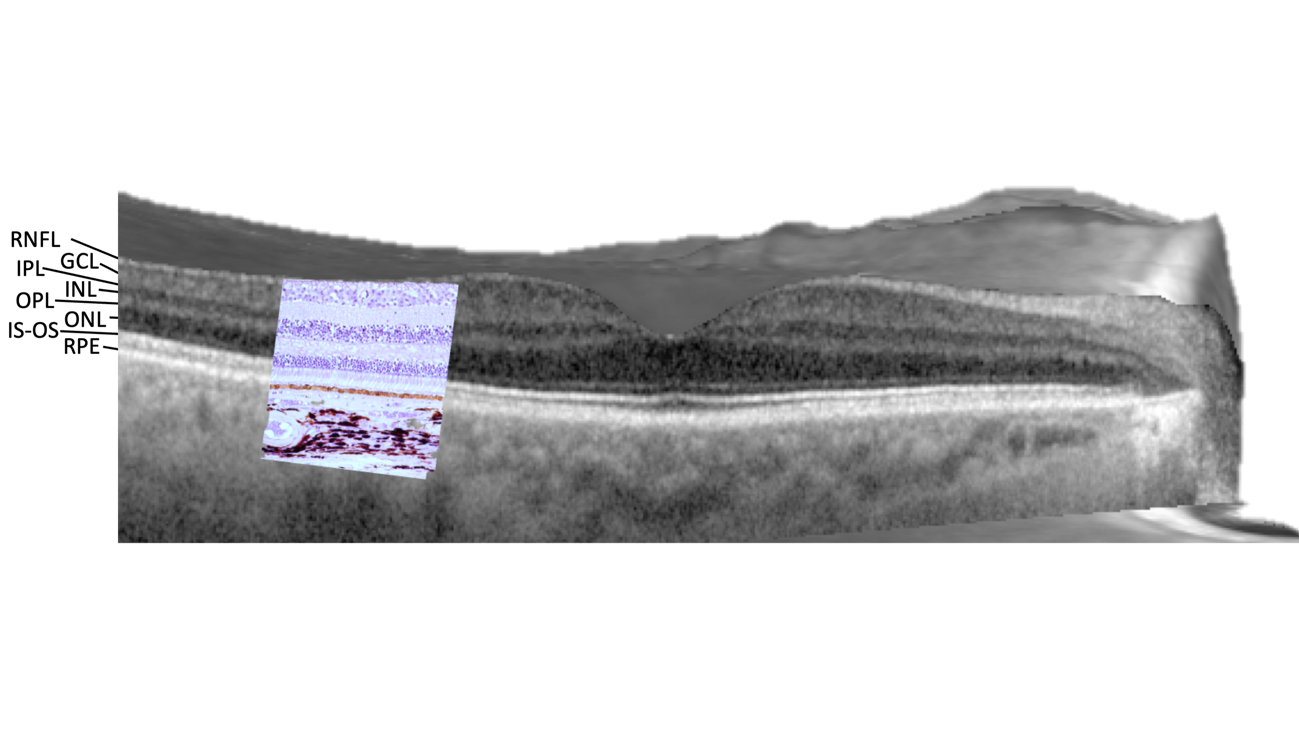


**Supplementary Figure 1**. OCT retinal representation of the layers. The image of the retina provided by OCT is shown in black and white and superimposed in colour on an image of a histological section of the retina stained with haematoxylin and eosin where the overlapping of the structures can be seen, showing the resolution for the observation of the retina by OCT.

The total retinal thickness and the thickness of each retinal layer in the macular zone were measured with Heidelberg software (Heidelberg, Germany, version 1.10.4.0). This measurement was checked by the same optometrist (IL-C) and manually modified if necessary. The thickness of the following retinal layers was analyzed: RNFL, retinal ganglion cell layer (GCL), inner plexiform layer (IPL), inner nuclear layer (INL), outer plexiform layer (OPL), outer nuclear layer (ONL) and retinal pigment epithelium (RPE). In the macula, the inner and outer rings were analyzed according to the standard Early Treatment Diabetic Retinopathy Study (ETDRS) macular grid (a foveal area of 1 mm in diameter, 1-3 mm around the fovea in the inner ring and 3-6 m for the outer ring) (E.T.D.R.S Group, 1991).

The pRNFL’s thickness was measured in six sectors (nasal, supero-nasal, infero-nasal, temporal, supero-temporal, infero-temporal), additionally obtaining an average of all sectors (Global) Supplementary Figure 2 .

**
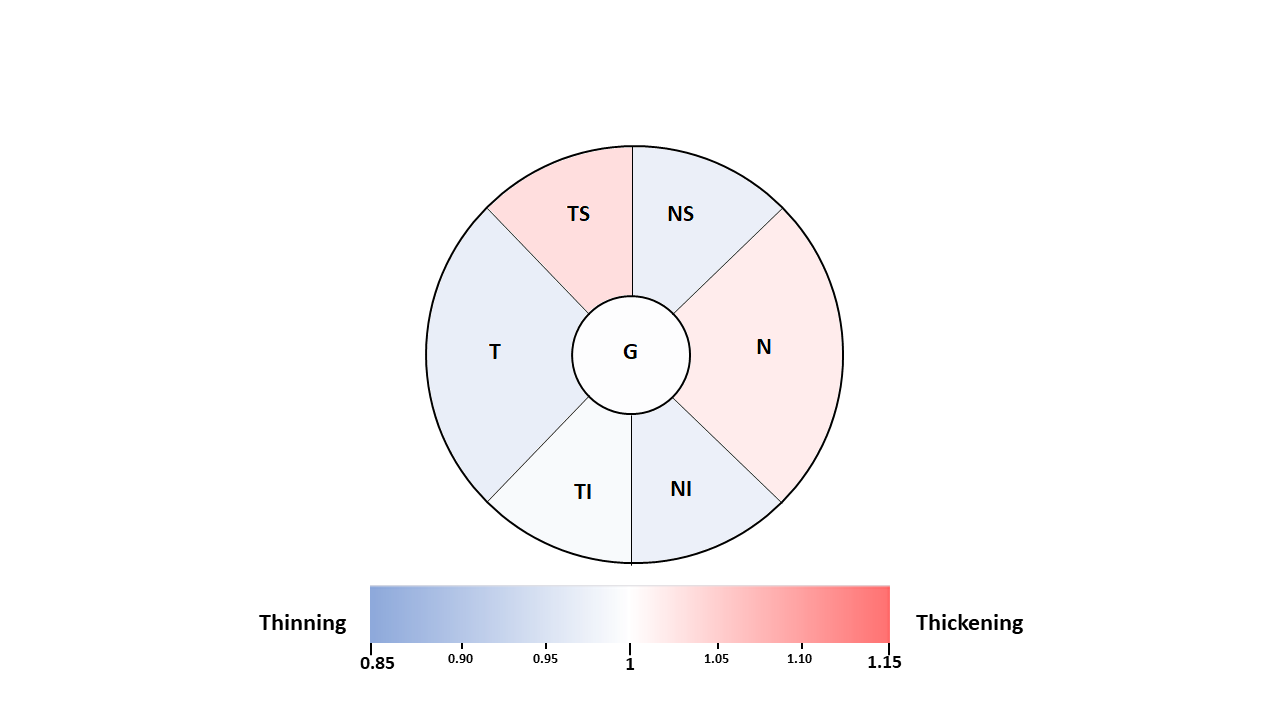
**

**Supplementary Figure 2.** Colorimetric representation of the peripapillary retinal nerve fiber layer thickness in the groups. FH+40-60ɛ4+ vs FH-40-60ɛ4- in the macular OCT sectors. In red, thickening; in blue, thinning. * p < 0.05. Wilcoxon rank sum test with continuity correction.

Good quality scans were considered with a minimum signal-to-noise ratio of 25 and an average of 16 B-scans. The pRNFL scans have a minimum signal-to-noise ratio of 20 and at least an average of 40 B-scans. According to the calibration provided by the manufacturers, measurements were given in µm. Both eyes of each subject were scanned, but only one eye of each participant was used in this study.

**Magnetoencephalography task**

The task consisted of a delayed-match to sample paradigm with faces as stimuli. For the present article, only the visual-related activity generated after the face presentation will be addressed. There were 128 trials, and, since each one contained two face presentations, there were 256 face-locked events in total. All faces were neutral, Caucasian, adult male and female faces on a gray noise background. To prevent cues that are not face specific, the hair and ears were removed. Stimuli were randomly presented and computerized using E-Prime software (version 1.2, Psychology Software Tools, Inc., Sharpsburg, PA). The stimuli were projected on a screen through a video projector (Panasonic PT-D7700E).


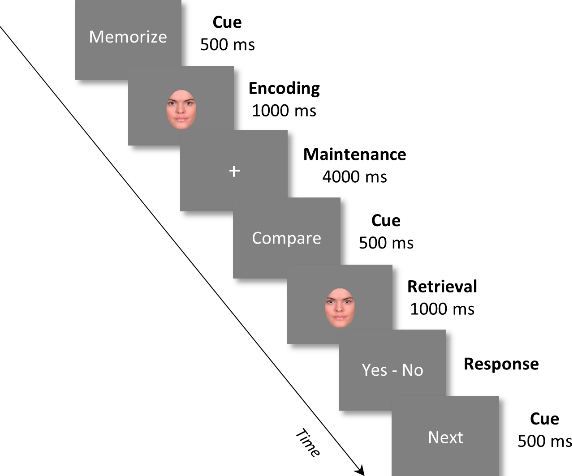


**Supplementary Figure 3**. Participants were asked to remember the encoding face during the maintenance period. Following delay, a probe face was presented and they compared whether it was the same or different to the encoded face.

The recording was performed at the Center for Biomedical Technology (Madrid, Spain), using a 306-sensor (102 magnetometers and 204 planar gradiometers) “Elekta Vectorview” MEG system (Elekta AB, Stockholm, Sweden) located inside a magnetically shielded room (VacuumSchmelze GmbH, Hanau, Germany). In addition to the MEG channels, two sets of bipolar EEG electrodes were used to record ocular and cardiac activity.

Four head position indication (HPI) coils were placed on the participants’ scalp; two on the mastoids and two on the forehead. The coils’ positions, together with the three anatomical landmarks (nasion, left and right preauricular points) and around 200 additional points of the participant’s head shape, were digitized using a Fastrack Polhemus system (Polhemus, Colchester, VT, USA). During the recording, the coils were fed to continuously determine the head position with respect to the MEG helmet.

MEG and EEG data were digitized with a sampling rate of 1000Hz, using an online band-pass anti-alias filter (0.1 to 330 Hz). To remove external noise and compensate for head movements inside the MEG scanner, a spatiotemporal expansion of the signal space separation (tSSS) method,1 implemented by Neuromag Software (MaxFilter version 2.2, correlation 0.90, time window 10s), was used.

**Magnetoencephalography preprocessing**

During the preprocessing of the MEG data, the analysts were blind to the conditions of the data. The magnetic time series were segmented into 2 s duration epochs (-500 ms to 1500 ms, relative to the beginning of the face presentation), using the period before 0 as baseline. Epochs that coincided with blinks or eye-movements, presented muscular activity, or were contaminated by other artifacts, were discarded. Afterwards, an independent component analysis-based procedure2 was applied to remove the contribution of the remaining eye-related, heart-related, and noise-related components. Given the redundancy between magnetometers and gradiometers data after tSSS, only magnetometer activity was employed in the following analyses.3

Magnetoencephalography source reconstruction

The source model was defined in the Montreal Neurological Institute (MNI) space, utilizing a homogeneous three-dimensional grid with sources spaced by 1 cm, resulting in 2459 source positions inside the head cavity. This source model was then linearly transformed into each participant’s individual MRI.

Each T1 image was segmented using unified segmentation (Penny et al. 2007) as provided by SPM12, and the resulting brain mask (i.e., union of white and gray matter and cerebrospinal fluid) was used to build an individual realistic single-shell head model. This head model, along with the individual source model, was employed to generate a lead field using a modified spherical solution.4

Time series of each source location were obtained using Linearly Constrained Minimum Variance (LCMV) beamformers5 as an inverse model. The beamformers were calculated using the covariance matrix of the data filtered between 0.2 and 35 Hz. Data were filtered using a pair of filters: a high pass Butterworth filter of second order and a low-pass finite impulse response (FIR) filter, built with Hamming window, of 1800th order. In order to avoid edge artifacts, data were filtered after adding 2 seconds of real data at each side, as padding.

1. Taulu S, Simola J. Spatiotemporal signal space separation method for rejecting nearby interference in MEG measurements. Phys Med Biol. 2006;51(7):1759-1768. doi:10.1088/0031-9155/51/7/008

2. Belouchrani A, Abed-meraim K. Using Second-Order Statistics. 1997;45(2):434-444.

3. Garcés P, López-Sanz D, Maestú F, Pereda E. Choice of magnetometers and gradiometers after signal space separation. Sensors (Switzerland). 2017;17(12):1-14. doi:10.3390/s17122926

4. Nolte G. The magnetic lead field theorem in the quasi-static approximation and its use for magnetoenchephalography forward calculation in realistic volume conductors. Phys Med Biol. 2003;48(22):3637-3652. doi:10.1088/0031-9155/48/22/002

5. Van Veen BD, Van Drongelen W, Yuchtman M, Suzuki A. Localization of brain electrical activity via linearly constrained minimum variance spatial filtering. IEEE Trans Biomed Eng. 1997;44(9):867-880. doi:10.1109/10.623056
